# Supplementary material for: Cytochrome P450 Expression and Chemical Metabolic Activity before Full Liver Development in Zebrafish
Source: Pharmaceuticals (Basel). 2020 Dec 11;13(12):456. doi: 10.3390/ph13120456 (PMC7763843; doi:10.3390/ph13120456)
Supplement: Supplementary file 1 [file pharmaceuticals-13-00456-s001.pdf]

pharmaceuticals

Supplementary materials

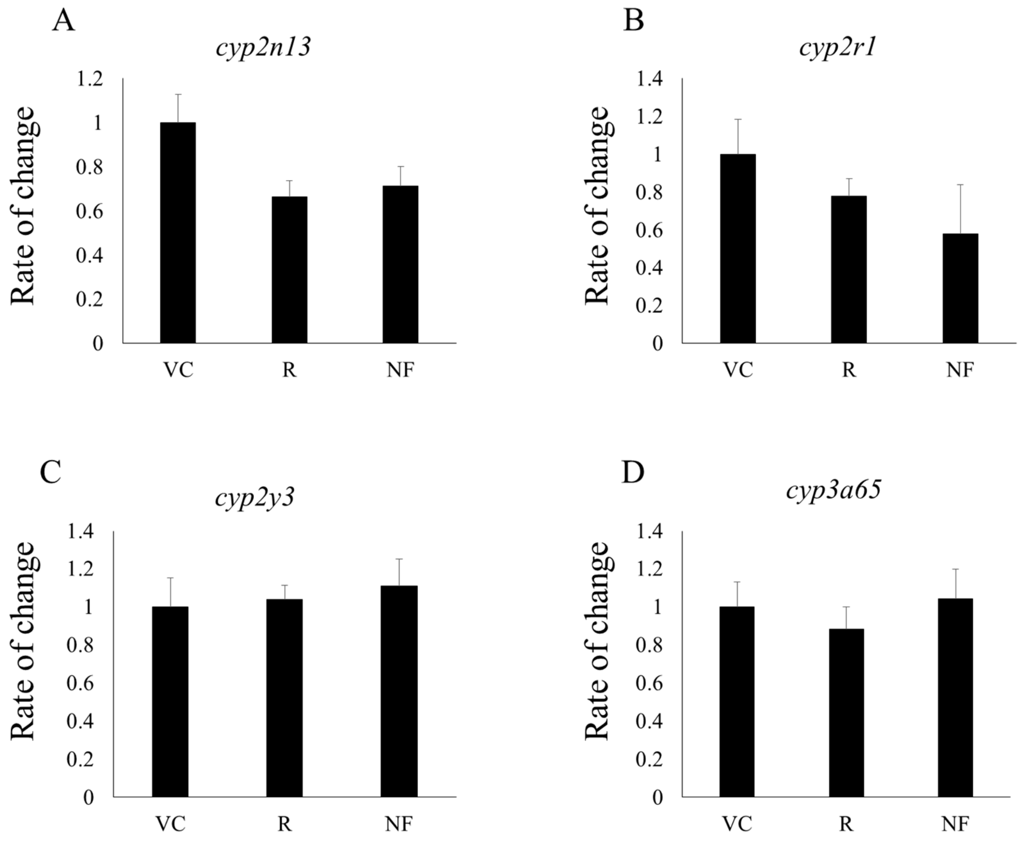

Figure 1. Effects of cytochrome P450 inducers on expression of a several CYP subtypes. Zebrafish embryos or larvae were exposed to 100 μM rifampicin (R) and 100 μM β-naphthoflavone (NF) from 26 hpf. VC means vehicle control (0.1% DMSO). cDNAs of larval zebrafish were prepared at 55 hpf for qPCR measurement of some CYP subtypes that showed positive signals by inducers with in situ hybridization.

Table S1. Primers used for quantitative real-time PCR

Primers	Sequence (5'-3')
cyp1a-F	TGCCGATTTCATCCCTTTCC
cyp1a-R	AGAGCCGTACTGATAGTGTC
cyp1b1-F	TCAGCTCGGTAACACTCCTC
cyp1b1-R	GGAATGAAGCAAAGTCGGGT
cyp1c1-F	GCGTAATTGAGCACGGGA
cyp1c1-R	CATCCATTGCATTGCTGTT
cyp1c2-F	TGGTGTAATGGATCATGCAG
cyp1c2-R	GCAGAAGCATCCAATTAAGC
cyp1d1-F	CGCCCTGACCTCTATACCTT
cyp1d1-R	GGATCAATCCCAGAGTCTCC
cyp2ad2-F	TGATCCAAACGAATGGGAAACC
cyp2ad2-R	TCACTTTGTTCTAACGTACAGAG
cyp2k18-F	CAACAGCAGCCTCTCTTTCTC
cyp2k18-R	AGCGTCTGATTGCACACAG
cyp2n13-F	GCTTCATCCCAAAGGGCA
cyp2n13-R	CAGATCTTGAACGGGTGTGG
cyp2r1-F	CCGAGCCTCTCAGCGAAATTGG
cyp2r1-R	GTCAACATCAGAGGTCAG
cyp2y3-F	TGGAATGGGACGCAAACGAA
cyp2y3-R	CACCAGAGAGCAGATGACGT
cyp3a65-F	GGTACTTCAAGAAGCTGGGC
cyp3a65-R	GGTCACCACATCCATACTGTACG
cyp3c1-F	TAGAACTCCGTGTTGAGGAGCGG
cyp3c1-R	GCCTTCCATCATAGATCCCCC
cyp3c2/3-F	GCAAGCGAGGCATTTTACA
cyp3c2/3-R	TAAACACATCCACAAGGCCA
cyp3c4-F	CATTTCTGTCTGTGACCTGG
cyp3c4-R	ATTTTAACTGGCTGCTCCGGG

Table S2. Primers used for preparation of probes for *in situ* hybridization

Primers	Sequence (5'-3')
cyp2n13-F	GCTTCATCCCAAAGGGCA
cyp2n13-R	CAGATCTTGAACGGGTGTGG
cyp2r1-F	CCGAGCCTCTCAGCGAAATTGG
cyp2r1-R	GTCAACATCAGAGGTCAG
cyp2y3-F	TGGAATGGGACGCAAACGAA
cyp2y3-R	CACCAGAGAGCAGATGACGT
Cyp3a65-F	GGTACTTCAAGAAGCTGGGC
Cyp3a65-R	GGTCACCACATCCATACTGTACG
